# Effective HAART reduces the incidence of high grade cervical neoplasia in HIV positive women

**DOI:** 10.1186/1742-4690-9-S1-P131

**Published:** 2012-05-25

**Authors:** Deborah Morris-Harris, Charmaine Miller-Spencer, Clara Jones, Song Zhang, James Luby

**Affiliations:** 1 Parkland Health and Hospital System, HIV Services, Dallas, Texas, USA; 2 Tufts University School of Medicine, Boston, Massachusetts, USA; 3 University of Texas Southwestern Medical School, Dallas, Texas, USA

## Introduction

Highly active antiretroviral therapy (HAART) has been shown to restore immunity and reduce the burden of Human Immunodeficiency Virus (HIV) in patients with HIV infections. Co-infection with HIV and the Human Papilloma Virus has been estimated to be as high as 51% in HIV positive women. Recently effective HAART has been shown to reduce the prevalence of HPV and intraepithelial lesions on Papanicolaou (pap) smear. It is unclear if HIV viral suppression can reduce the incidence of high grade cervical neoplasia .

## Materials and methods

A retrospective cohort of 1090 women, in care from 2005 through 2008, had 389 referrals to colposcopy clinic. 142 women mean age of 31.2 (range 17-54); 66.2% Non -Hispanic black,9.9% Non- Hispanic white, and 23.9% Hispanic with HIV, median baseline CD4 283(IQR 87.75-508) had a colposcopy and a second procedure, either colposcopy or excisional biopsy after a baseline abnormal Pap smear. Follow-up biopsies were performed with a median of 11.9 months (IQR 4.4-22.5) There were no significant baseline differences in clinical or demographic parameters between patients who were suppressed with VL.

## Results

Of 125 cases in which HAART was started before the first colpsocopy; 26 had a normal colposcopy and 48 had CIN1. From the normal and low grade group, 19 women developed high grade CIN (7, suppressed and 12, not suppressed.) Effective HAART reduced the risk of CIN2-CIS on a second biopsy by 83.2% (AHR 0.168; C.I. = 0 .057 -.498; p< 0.001) adjusted for age at diagnosis, race, smoking and baseline CD4. Higher baseline CD4 was associated with a reduced risk of high grade neoplasia (AHR=0.996; C.I. 0.993-0.999; p

## Conclusions

Reducing HIV viral load and preserving CD4 cells early in the course of HIV and HPV co-infection decreases the incidence of high grade cervical neoplasia.

